# Role of Endoplasmic Reticulum Stress in Atherosclerosis and Its Potential as a Therapeutic Target

**DOI:** 10.1155/2020/9270107

**Published:** 2020-09-09

**Authors:** Shengjie Yang, Min Wu, Xiaoya Li, Ran Zhao, Yixi Zhao, Longtao Liu, Songzi Wang

**Affiliations:** ^1^Guang'anmen Hospital, China Academy of Chinese Medical Sciences, Beijing, China; ^2^Beijing University of Chinese Medicine, Beijing, China; ^3^Xiyuan Hospital, China Academy of Chinese Medical Sciences, Beijing, China

## Abstract

Endoplasmic reticulum (ER) stress is closely associated with atherosclerosis and related cardiovascular diseases (CVDs). It occurs due to various pathological factors that interfere with ER homeostasis, resulting in the accumulation of unfolded or misfolded proteins in the ER lumen, thereby causing ER dysfunction. Here, we discuss the role of ER stress in different types of cells in atherosclerotic lesions. This discussion includes the activation of apoptotic and inflammatory pathways induced by prolonged ER stress, especially in advanced lesional macrophages and endothelial cells (ECs), as well as common atherosclerosis-related ER stressors in different lesional cells, which all contribute to the clinical progression of atherosclerosis. In view of the important role of ER stress and the unfolded protein response (UPR) signaling pathways in atherosclerosis and CVDs, targeting these processes to reduce ER stress may be a novel therapeutic strategy.

## 1. Introduction

Atherosclerosis is a critical pathological factor in the development of cardiovascular diseases (CVDs), which are a serious threat to human health and are one of the major causes of death worldwide [1, 2]. The pathogenesis of atherosclerosis is a complex process involving a variety of metabolic and signaling pathways. Several known risk factors include metabolic disorders, dyslipidemia, hyperglycemia, hypertension, and elevated homocysteine (Hcy) levels [3–5]. The formation and development of atherosclerotic lesions involve the pathological processes of lipid accumulation in the arterial wall, local inflammatory processes, and endothelial dysfunction [6, 7]. Increasing evidence indicates that endoplasmic reticulum (ER) stress signaling pathways play important roles in atherosclerosis and its related CVDs. The ER is an organelle in eukaryotic cells that is important for protein synthesis, folding, and transport; lipid synthesis; and calcium homoeostasis [8]. Various pathological factors, such as hyperlipidemia, oxidative stress, and calcium imbalance, may lead to perturbations in ER homeostasis, which are manifested as the accumulation of unfolded or misfolded proteins in the ER lumen, causing ER stress [9, 10]. Chronic ER stress is associated with the development of atherosclerosis through a variety of mechanisms. This pathological process may involve ER stress mediating the activation of inflammatory response mechanisms and apoptotic signaling pathways. This affects lipid metabolism, leading to cell dysfunction and affecting the formation and stability of atherosclerotic plaques, all of which are important conditions for atherosclerosis development [11–14]. At the same time, considering the important roles of ER stress signaling pathways and their mediation of multiple pathologic pathways, targeting ER stress pathways may be a promising therapeutic strategy for atherosclerosis and CVDs. In this review, we discuss the role of ER stress in atherosclerosis and its potential as a therapeutic target.

## 2. ER Stress and Unfolded Protein Response (UPR)

In order to protect ER functional integrity and cell homeostasis, UPR, an evolutionarily conserved signaling cascade, is activated upon ER stress [15, 16]. The main mechanism is known to involve activation of three stress sensors located on the ER membrane: protein kinase RNA-like endoplasmic reticulum kinase (PERK), inositol-requiring enzyme 1 (IRE1), and activating transcription factor 6 (ATF6) [17]. In the unstressed state, the UPR remains inactive through the binding of the 78 kDa glucose-regulated protein (BiP/GRP78) to the lumen domains of the three pivotal ER transmembrane proteins mentioned above [18]. When unfolded or misfolded proteins accumulate in the ER lumen, BiP/GRP78 dissociates to assist in the folding process, thus initiating the UPR signaling cascade. GRP78 dissociation is the current mainstream view of UPR activation, but other unknown mechanisms may also be involved [19].

As an initial response to ER stress, the UPR regulates and restores ER function mainly by blocking protein translation, upregulating ER chaperone proteins, facilitating protein folding, and guiding misfolded proteins into the correct degradation pathway [8]. PERK is activated by autophosphorylation after dissociation from BiP/GRP78. At the early stage of the ER stress response, the UPR first reduces protein overload through activated PERK (phospho-PERK)-mediated eukaryotic initiation factor 2*α* (eIF2*α*) phosphorylation, which results in translational attenuation and subsequent alleviation of ER stress. IRE1 is also activated after separation from BiP/GRP78, and its site-specific endoribonuclease function then regulates the specific mRNA splicing of X-box binding protein 1 (XBP1) to form XBP1s, which is subsequently translated to active XBP1 protein [20]. The genes that are upregulated by activated XBP1 are related to ER chaperones (such as GRP78/94) and promote protein folding and misfolded protein degradation [21]. In addition, activated XBP1 can regulate the transcription of components involved in the ER-associated degradation process, thus reducing misfolded protein load. When ATF6 is released by BiP/GRP78, it is translocated to the Golgi and further becomes activated by proteolytic cleavage [22]. This is followed by ATF6 translocation to the nucleus, stimulating the expression of genes involved in the adaptive stress response, including GRP78 and XBP1 [23]. During the UPR process, phosphorylation of eIF2*α* regulates the translation of certain mRNAs including activating transcription factor 4 (ATF4). ATF4, ATF6, and XBP1 are associated with the expression of C/EBP-homologous protein (CHOP), a widely studied biomarker involved in the ER stress-associated apoptosis signaling pathway [24, 25]. When the UPR fails to normalize ER function, long-term ER stress causes activation of apoptosis and inflammatory response pathways.

## 3. Proatherogenic Effects of ER Stress in Different Cell Types

### 3.1. ER Stress in Endothelial Cells (ECs)

The theory of the injury response of vascular endothelial cells (VECs) is one of the most recognized pathogenesis models of atherosclerosis. Endothelial dysfunction plays a role as an initiating factor in atherosclerosis. Atherosclerosis occurs most often in areas of turbulent blood flow, such as vessel bending or branching [26]. ECs experience a constant strain of blood flow and are particularly susceptible in these areas. Evidence from nonatherosclerotic swine suggested that the ER stress markers IRE1, XBP1, and ATF6 are activated in ECs in atherosclerotic-susceptible areas of the aorta [27]. Recently, studies have found that disturbed blood flow with low shear stress (SS), a major atherogenic factor leading to EC dysfunction, can directly induce ER stress in ECs, thus exerting critical effects on the progression of atherosclerosis [28]. For in vitro cultured ECs, atherogenic SS preferentially upregulated the expression of the UPR regulator GRP78 through a p38- and integrin alpha2beta1-dependent mechanism before atheroprone lesion development, which reflects a potential atheroprotective and compensatory response to ER stress [29]. Nonetheless, atherogenic SS activates ER stress to promote an endothelial proinflammatory phenotype. Bailey et al. found that SS mechanoregulates the inflammatory response of human aortic ECs through activating the transcription factor XBP1 via ER stress, during which a temporal, SS-regulated rise in p38 phosphorylation activates XBP1 nuclear translocation and promotes the highest expression of vascular cell adhesion protein 1 (VCAM-1). In summary, a possible mechanism is that SS sensitizes the ECs to cytokine-induced ER stress, thereby modulating inflammation that promotes atherosclerosis [30, 31]. In addition, SS induces human aortic EC apoptosis, the mechanism of which involves ER stress mediated by the interleukin-1 receptor-associated kinase 2 (IRAK2)/CHOP signaling pathway [32]. It is worth noting that these effects can all be ameliorated by ursodeoxycholic acid (UDCA). In a disturbed flow-induced atherosclerosis mouse model, UDCA effectively reduced ER stress, evidenced by decreased expression of XBP1 and CHOP in ECs, and it inhibited the inflammatory response and apoptosis of ECs caused by disturbed flow, thus suppressing the formation of atherosclerotic plaques [28].

Homocysteine (Hcy) is a crucial atherorelevant inducer that activates pathological ER stress in ECs. Studies have shown that Hcy-induced ER stress can cause specific changes in gene expression and programmed cell death in human umbilical vein endothelial cells (HUVECs) [33–35]. Under hyperhomocysteinemia (HHcy), ER stress may lead to vascular inflammation and endothelial dysfunction. Mechanistic studies have revealed that there is a reactive thiol group in Hcy that can cause functional changes in critical proteins, which maintain normal vascular function, through disulfide exchange with cysteine residues in these proteins [36]. While intracellular Hcy concentration was increased, this Hcy-induced protein modification also occurred in the ER, secreted proteins and membrane proteins [3]. Consistent with these data, the expression of RTP (reducing agent and tunicamycin-responsive protein), which was originally identified as an Hcy-responsive gene product, was induced in HUVECs under conditions causing ER stress [37]. The proatherogenic effect of Hcy-induced ER stress also occurs in other lesion cell types in addition to the ECs.

During atherosclerosis, modified (such as oxidized, glycosylated, or phospholipolyzed) low-density lipoprotein (LDL) disrupts ER calcium metabolism, thus inducing endothelial UPR and oxidative stress, the latter of which inhibits the sarcoplasmic/endoplasmic reticulum Ca^2+^-ATPase (SERCA). Studies have shown that phospholipolyzed LDL induces inflammatory responses in ECs via ER stress [38]. In addition, oxidized- (ox-) LDL was shown to induce inflammatory pathology modifications in ECs, leading to EC injury through inflammasome activation mediated by apoptosis signal-regulating kinase 1 (ASK1)/NOD-, LRR-, and pyrin domain-containing protein 3 (NLRP3) via ER stress [39]. ox-LDL also mediated apoptosis in VECs mainly through the PERK/eIF2*α*/CHOP signaling pathway of ER stress [40]. ER stress and apoptosis induced by ox-LDL could be inhibited by simvastatin [41]. In conclusion, modified LDL plays an important role in ER stress-mediated endothelial dysfunction, inflammation, and apoptosis in atherosclerotic vessels.

**Figure 1:**
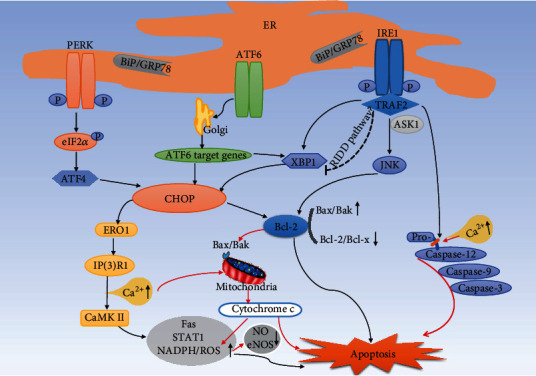
ER stress-induced apoptosis in lesional macrophages and ECs. When the UPR fails to normalize ER function, prolonged ER stress will activate the proapoptotic pathway and eventually induce apoptosis. This mainly involves the following mechanisms in macrophages: (1) CHOP mediates activation of the ERO1/IP3R1/CaMK II calcium signaling pathway and its downstream apoptotic pathway. (2) CHOP regulates the Bcl-2 family, which controls the balance between the proapoptotic and antiapoptotic signals, thus controlling apoptosis. (3) The IRE1/TRAF2 complex interacts with ASK1 to induce JNK activation and then regulates Bcl-2 family members to promote cell apoptosis. (4) Calcium homeostasis imbalance and IRE1/TRAF2 activate the caspase-12 cascade, which eventually induces apoptosis. (5) The coinduction of low-dose ER stressors and atherorelevant second hits, such as the activation of PRRs, led to macrophage apoptosis. In ECs (red arrows), CHOP-mediated imbalance of the Bcl-2 family activated proapoptotic proteins on the mitochondrial membrane to induce the release of cytochrome c, leading to subsequent mitochondrial-dependent apoptosis. This process, together with calcium homeostasis imbalance, leads to decreased mitochondrial function and increased levels of NADPH and ROS in ECs under the pathological conditions of atherosclerosis, thus causing apoptosis and vascular endothelial dysfunction. CaMK II: calcium/calmodulin-dependent protein kinase II; STAT1: signal transducer and activator of transcription 1; PRRs: pattern recognition receptors.

ER stress acts as a defense mechanism that enables cells to respond to harmful stimuli. However, when UPR fails to normalize ER function, prolonged ER stress will activate the proapoptotic pathway and eventually induce apoptosis, which has been recognized as an important pathologic factor of atherosclerosis and many CVDs [6, 42]. The activation of apoptosis pathways mediated by the ER stress-mitochondrial cascade may be a crucial mechanism of EC apoptosis (Figure 1). Under ER stress, it was found that CHOP-mediated imbalance of the Bcl-2 family activated proapoptotic proteins on the mitochondrial membrane, inducing cytochrome c release and causing subsequent mitochondrial-dependent apoptosis [43]. This process, together with imbalanced calcium homeostasis, leads to decreased mitochondrial function and increased levels of NADPH and reactive oxygen species (ROS) in ECs under the pathological conditions of atherosclerosis [44–46]. Studies have indicated that NADPH and ROS inhibit the nitric oxide (NO) production and activity of endothelial nitric oxide synthase (eNOS), causing enhanced oxidative stress and vascular endothelial dysfunction [47–49]. Under ER stress, cytosolic Ca^2+^ overload activated the inactive proenzyme procaspase-12 to form caspase-12 in the ER membrane of ECs; caspase-3 and apoptosis were eventually activated in these cells along with calpain-mediated caspase-9 activation [25]. In the ECs of apolipoprotein E (ApoE)^−/−^ atherosclerosis model mice, antiapoptotic Bcl-2 was significantly decreased while caspase-3 was significantly increased [50]. Another previous study in HUVECs showed that silica nanoparticles induced ER stress-related activation of the IRE1*α*/c-Jun N-terminal kinase (JNK) pathway, CHOP, and caspase-12, accompanied by increased proapoptotic Bax, reduced antiapoptotic Bcl-2, and upregulated expressions of caspase-9, caspase-3, and cytochrome c [51].

### 3.2. ER Stress in Macrophages

During atherosclerosis progression in ApoE^−/−^ mice fed high-fat diet (HFD), it was initially demonstrated that macrophages were particularly prominent cells undergoing ER stress in atherosclerotic lesions [52]. ER stress plays a key role in the death of advanced lesional macrophages. Evolving mechanistic studies performed on in vitro-cultured macrophages and in vivo mouse models of atherosclerosis supported the fact that ER stress-induced macrophage apoptosis is a crucial event in inflammatory necrotic core generation and contributes to the instability of advanced atherosclerotic plaques, laying the foundation for subsequent plaque rupture [6, 14, 53, 54].

CHOP is the most extensively studied biomarker involved in ER stress-related apoptosis signals [25]. A significant relationship between CHOP expression and lesional apoptosis has been revealed in human atherosclerosis stages; that is, advanced and vulnerable plaques show enhanced CHOP expression and apoptosis [55]. Research with cultured macrophages found that under atherosclerotic conditions, expression of CHOP increased with the development of ER stress, and eventually CHOP and its downstream apoptosis signaling pathways were activated; this is one of the most common mechanisms of ER stress-mediated apoptosis in macrophages [13, 56]. The specific molecular signaling pathways involved are shown in Figure 1. Mechanistic studies have suggested that activation of the CHOP-mediated apoptosis pathway is associated with calcium signaling. The CHOP transcriptional target endoplasmic reticulum oxidoreductin 1 (ERO1) overoxidized the ER lumen, leading to the activation of inositol 1,4,5-trisphosphate receptor type 1 (IP3R1) and subsequent formation of disulfide bonds in the IP3R1 luminal loop [57–59]. This process eventually enhanced the calcium channel activity of IP3R1, resulting in increased calcium release. Increased cytoplasmic calcium led to the activation of calcium/calmodulin-dependent protein kinase (CaMK) II, consequently activating many proapoptotic pathways including the death receptor Fas, apoptosis pathways mediated by mitochondria, a proapoptotic pathway involving signal transducer and activator of transcription 1 (STAT1), and the NADPH/ROS pathway [6, 44]. Notably, activation of the CHOP-mediated apoptotic signaling pathway regulates the Bcl-2 family, a crucial apoptotic factor which controls the balance between proapoptotic (known members Bax and Bak) and antiapoptotic (known members Bcl-2 and Bcl-x) signals. Studies have shown that CHOP-mediated macrophage apoptosis promotes atherosclerotic plaque rupture, which is induced in a CHOP-Bax pathway-dependent manner [6, 14].

In addition to CHOP-related pathways, another mechanism of ER stress-induced apoptosis is activation of the IRE1-mediated apoptosis pathway. IRE1 interacts with tumor necrosis factor (TNF) receptor-associated factor-2 (TRAF2), and this complex is closely related to the signal transduction factor ASK1, which activates JNK and then regulates Bcl-2 family members to promote cell apoptosis [6, 60, 61]. In addition, during the interaction of IRE1*α*/TRAF2/ASK1 in ER stress, the association of TRAF2/procaspase-12 activates caspase-12 and eventually induces apoptosis [25]. Evidence has also been reported to support the IRE1-dependent degradation of ER-related mRNAs through regulated IRE1*α*-dependent decay (RIDD) under high levels of ER stress signals, leading to cell apoptosis [13, 62, 63].

It is known that tunicamycin, thapsigargin (SERCA inhibitor), and high cellular levels of unesterified cholesterol, oxidative stress, and peroxynitrate, which are atherorelevant ER stressors, can lead to prolonged activation of the UPR [64, 65]. Another possible mechanism of macrophage apoptosis revealed by previous studies is coinduction by low-dose ER stressors and atherorelevant second hits, such as the activation of pattern recognition receptors (PRRs) [6, 65, 66]. PRRs include toll-like receptors (TLRs) and scavenger receptors (such as CD36, a type A scavenger receptor (SRA)). This view is supported by studies by Seimon et al., which demonstrated that atherogenic lipids, including oxidized phospholipids, ox-LDL, and lipoprotein (a), act together with the participation of CD36 and TLR2 to trigger apoptosis of macrophages undergoing ER stress [67]. Evidence from a mouse model of hypertriglyceridemia-induced atherosclerosis indicated that TRLs enhance macrophage ER stress and oxidative stress in a dose-dependent manner [68]. In addition, oxidized high-density lipoprotein (ox-HDL) activated the TLR4-dependent CHOP pathway by enhancing oxidative stress, thus inducing the apoptosis of macrophages under ER stress [69]. Minimally modified LDL induced the accumulation of free cholesterol in the ER, which in turn stimulated ATF6- and IRE1-mediated ER stress in RAW264.7 macrophages; this process may also be mediated by TLR4 [70].

### 3.3. ER Stress in Smooth Muscle Cells

In recent years, studies on the role of ER stress in vascular smooth muscle cells (VSMCs) in atherosclerosis have been increasing. In a progeria model of ApoE^−/−^ mice, ER stress and the UPR were identified as drivers of VSMC death, which further accelerated atherosclerosis [71]. ER stress-induced apoptosis of VSMCs could result in a thinned protective collagen cap, which might be an important mechanism for the transition of advanced atherosclerotic plaques from stable to vulnerable [72]. Protecting VSMCs from plaque apoptosis has been a potentially crucial therapeutic target for stabilization of atherosclerotic plaques. An example is the highly expressed selenoprotein S (SelS), which was significantly correlated with atherosclerotic CVD in epidemiological studies; SelS might protect VSMCs from apoptosis by suppressing ER stress and oxidative stress [73]. In advanced atherosclerosis, CHOP is known to promote macrophage apoptosis, but its role in VSMCs in atherosclerosis has not been fully studied. Zhou et al. found in their study that CHOP expression in VSMCs induces cell proliferation in atherosclerotic lesions by downregulating Krüppel-like factor 4, which is a pivotal suppressor of VSMC proliferation [74]. Phenotypic transformation of VSMCs plays an important role in atherosclerosis, and the ER stressor Hcy is related to this process to some extent. HHcy usually can be induced by a high methionine diet (HMD). A study showed that HMD led to significant activation of the ATF6/homocysteine-inducible endoplasmic reticulum protein (HERP) arm of ER stress in low-density lipoprotein receptor (LDLR)^−/−^ mice, which induced phenotypic transformation of VSMCs; knockdown of HERP inhibited this process, attenuating HHcy-mediated atherosclerosis [75]. In addition, Hcy activated sterol regulatory element-binding protein 2 (SREBP-2) in VSMCs cultured in vitro, leading to increased intracellular lipid accumulation [76, 77]. Besides Hcy, a novel ER stress regulator, GRP78-regulated protein interaction protein (Gipie), which is involved in VSMC ER stress and affects VSMC survival and neointimal formation after vascular injury, was reported by Noda et al. Gipie knockdown caused increased JNK phosphorylation and apoptotic cell numbers under ER stress [78]. Vascular calcification is an important characteristic of hypertension and atherosclerosis. A recent study found that death-associated protein kinase 3 (DAPK3) regulates the calcification of VSMCs via 5′ adenosine monophosphate-activated protein kinase- (AMPK-) mediated ER stress signaling. DAPK3 knockout inhibited the expression of ER stress-related proteins and delayed the phenotypic switching of VSMCs into osteogenic cells, a crucial process for vascular calcification [79].

### 3.4. ER Stress-Induced Inflammation in Atherosclerosis

**Figure 2:**
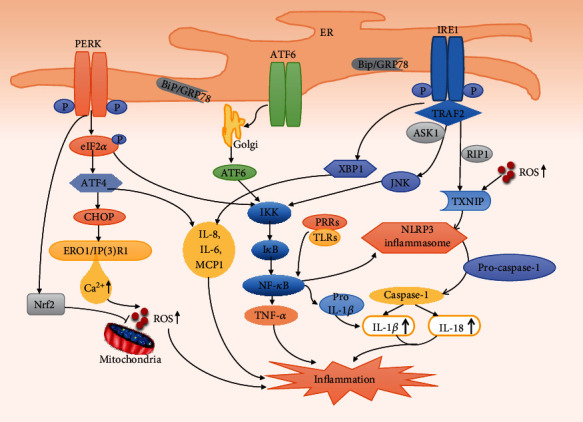
ER stress-induced inflammation in macrophages and ECs. Under ER stress conditions in macrophages and ECs, the three ER stress sensors, PERK, IRE1, and ATF6, can all activate the NF-*κ*B pathway and induce specific inflammatory responses. In addition, IRE1 induces the elevation of TXNIP, thereby activating the NLRP3 inflammasome, which in turn promotes caspase-1 activation and IL-1*β* and IL-18 secretion and an inflammatory response. RIP1 may be involved in this activation process. Under ER stress, increased calcium release leads to increased intracellular production of ROS, which is partly attenuated by the PERK-mediated transcription factor Nrf2 antioxidant program, but increased ROS levels may still lead to inflammation, contribute to NLRP3 activation to some extent, and promote ER dysfunction. XBP1s and ATF4 induce the production of inflammatory cytokines IL-8, IL-6, MCP1, and TNF-*α*. These all lead to inflammation and are involved in the development of atherosclerosis. ERO1: endoplasmic reticulum oxidoreductin 1; IP3R1: inositol 1,4,5-trisphosphate receptor type 1; Nrf2: nuclear factor erythroid 2-related factor-2; ROS: reactive oxygen species; MCP1: monocyte chemoattractant protein 1; TRAF2: tumor necrosis factor receptor-associated factor-2; ASK1: apoptosis signal-regulating kinase 1; JNK: c-Jun N-terminal kinase; I*κ*B: inhibitor of nuclear factor-*κ*B; IKK: I*κ*B kinase; NF-*κ*B: nuclear factor-*κ*B; RIP1: kinase receptor-interacting protein 1; TXNIP: thioredoxin-interacting protein.

Atherosclerosis is a chronic inflammatory disease in which inflammatory signaling pathways are involved in many stages throughout its progression [80–82]. Increasing evidence suggests that ER stress is associated with inflammatory signaling pathways through multiple mechanisms and it plays a significant role in atherosclerotic CVDs [83]. Specifically, the three ER stress sensors, PERK, IRE1, and ATF6, can all induce specific inflammatory responses via the UPR under challenging cellular ER stress conditions particularly in macrophages and ECs (Figure 2).

PERK-mediated attenuation of translation leads to phosphorylation of the inhibitor of nuclear factor-*κ*B (NF-*κ*B), I*κ*B, in which I*κ*B kinase (IKK) is involved. Subsequently, NF-*κ*B is released and translocated to the nucleus, which activates the expression of genes involved in downstream pathways of inflammation, such as those that encode the cytokines TNF-*α* and interleukin- (IL-) 1 [83, 84]. The NF-*κ*B-IKK pathway is a key regulator of inflammatory induction, and IRE1*α* can lead to activation of this pathway. Activated IRE1*α* recruits TRAF2, which interacts with JNK and IKK, and subsequently phosphorylates and activates downstream inflammatory pathways [85]. IRE1*α* siRNA attenuated inflammation and downregulated the expression of I*κ*B and phosphorylation of IKK, which suppressed the degradation of I*κ*B and nuclear translocation of NF-*κ*B p65 in RAW264.7 macrophages treated with angiotensin II [86]. Emerging evidence suggests that the NLRP3 inflammasome, a polyprotein complex produced by activation of PRRs, plays an important role in ER stress and the development of atherosclerosis [87, 88]. Mechanistic studies have shown that activation of the NLRP3 inflammasome contains two independent signals in macrophages. The first is the activation of PRR by an initial priming signal, which induces proinflammatory NF-*κ*B signaling [89]; the transcription factor NF-*κ*B translocates to the nucleus and induces transcriptional upregulation of pro-IL-1*β* (IL-1*β* precursor) and NLRP3. The second is NLRP3 activation to induce inflammasome assembly [89, 90]. Under ER stress, a possible pathway is that IRE1 induces elevation of thioredoxin-interacting protein (TXNIP), which activates the NLRP3 inflammasome [91]. The activated NLRP3 inflammasome converts pro-caspase-1 into activated caspase-1, which in turn promotes the secretion of IL-1*β* and IL-18 and leads to an inflammatory response [92–94]. A recent study showed that ER stress-induced inflammasome activation required the kinase, receptor-interacting protein 1 (RIP1), and suppression of RIP1 kinase activity or RIP1 knockdown remarkably reduced caspase-1 cleavage and IL-1*β* secretion induced by ER stress in J774A.1 macrophages and bone marrow-derived macrophages [95]. In addition to involvement in the inflammatory response, excessive production of IL-1*β* aggravates ER stress-mediated EC apoptosis through the IRAK2/CHOP signaling pathway, thereby promoting atherosclerosis [32].

Under ER stress, the generation of intracellular ROS usually increases and even reaches toxic levels, partly by increasing the release of calcium to increase the production of mitochondrial ROS [46, 96]. Although the UPR resists ROS increases through activation of the PERK-mediated antioxidant program via the transcription factor, nuclear factor erythroid 2-related factor-2 (Nrf2), and neutralization of toxic substances, chronic ER stress still leads to increased ROS levels that may lead to an inflammatory response [97]. The increased ROS in turn contributes to accelerated ER dysfunction and directly participates in protein secretion, folding, and degradation, thereby forming a connection between ER stress and oxidative stress [98]. Evidence from HFD-fed mice showed that ER stress-induced NLRP3 activation caused by palmitate stimulation is mediated by the ROS-TXNIP pathway [99]. Moreover, activation of AMPK inhibited this process and improved mitochondrial morphology and ER stress-associated endothelial dysfunction [99, 100].

The third branch of the UPR, the ATF6 pathway, also activates the NF-*κ*B pathway [101]. In addition, XBP1s and ATF4 induced the production of the inflammatory cytokines IL-8, IL-6, monocyte chemoattractant protein 1 (MCP1), and TNF-*α* in human ECs [83]. TLRs are host defense receptors that can recognize invading pathogens [102]. When ER stress and TLR signaling activation occur concomitantly, spliced XBP1 is also involved in production of the interferon cytokine family (IFN-*α*, IFN-*β*), which is essential for the body's defense [83]. ER stress inducers could increase the expression of TLR2 in epithelial cells. Overexpression and knockdown experiments indicated that ATF4 plays an important role in this process [103].

## 4. Therapeutic Potential of Regulating ER Stress Modulators for Atherosclerosis

### 4.1. Chemical Chaperones

The use of chemical chaperones is one of the possible treatments for ER stress mitigation. As small molecular factors, chemical chaperones can reduce the ER protein load under stress by nonselectively stabilizing unfolded proteins and promoting their normal folding [8]. 4-Phenylbutyric acid (4-PBA) and tauroursodeoxycholic acid (TUDCA) are two FDA-approved chemical chaperones that can be used in humans. ER stress is one of the potential causes of monocyte dysfunction in atherosclerosis. Treatment with 4-PBA could alleviate ER stress and apoptosis induced by glucolipotoxicity in human THP-1 monocytes [104]. Endothelial dysfunction is considered to be an important manifestation of atherosclerosis, and suppression of ER stress by 4-PBA could alleviate endothelial dysfunction [105]. Studies have shown that inhibition of ER stress by 4-PBA can attenuate glucosamine-induced proapoptotic, proinflammatory, and prothrombotic states in HUVECs [106] and can alleviate the effect of ox-LDL on the cholesterol efflux, apoptosis, ROS production, and inflammation of ECs [39]. 4-PBA also blocked the dephosphorylation of Akt and eNOS [107] and mitigated the apoptosis of macrophage-derived foam cells induced by ox-LDL [108]. Wang et al. found that the modulation of ER stress by 4-PBA mainly involved upregulation of the negative immunoregulatory molecules IL-35, IL-10, and forkhead box P3 (FOXP3), as well as accompanied increases in regulatory T cells (Tregs) in ApoE^−/−^ mice [109]. 4-PBA administration also inhibited the upregulation of CD36, GRP78, and phospho-IRE1 in macrophages from atherosclerotic lesions and peritoneal macrophages in ApoE^−/−^ mice [110]. These results showed the beneficial effect of 4-PBA on atherosclerosis by inhibition of ER stress and the restoration of ER function, which opposed the harmful effects of toxic lipids promoting atherosclerotic lesions [111]. TUDCA is another chemical chaperone that inhibits ER stress. It was found to suppress ER stress-induced apoptosis by decreasing calcium efflux, blocking the activation of caspase-12, and activating phosphoinositide 3-kinase (PI3K) survival signaling cascades [8, 112]. Oral administration of TUDCA effectively reduced ER stress and alleviated aortic lesion development in AMPK*α*2^−/−^ mice [113]. Ursodeoxycholic acid (UDCA), a hydrophilic endogenous bile acid, is the precursor form of TUDCA before conjugation with taurine. In a mouse model of disturbed flow-induced atherosclerosis, UDCA was found to inhibit the formation of atherosclerotic plaques by inhibiting ER stress and attenuating inflammatory responses, as evidenced by decreased expression of XBP1 and CHOP and reduced adhesion molecule levels in ECs [28].

Another chemical chaperone, SRT1720, eliminated glucosamine-induced ER stress and reversed its influence on apoptosis and procoagulant/proinflammatory pathways in HUVECs. This action of SRT1720 was modulated by its ability to regulate raptor acetylation, thereby suppressing mammalian target of rapamycin complex 1- (mTORC1-) dependent protein synthesis and reducing ER overload [106]. In summary, chaperones, especially chemical chaperones, may be promising treatments for atherosclerosis.

### 4.2. Inhibition of Upregulated Signaling Pathways in ER Stress

Targeted inhibition of the three primary branches of the UPR (PERK/eIF2*α*, ATF6, and IRE1) can attenuate ER stress and thus exert a protection effect. 2-Aminopurine (2-AP) is a phosphorylation inhibitor of eIF2*α*, and treatment with 2-AP significantly downregulated GRP78 and phosphorylated eIF2*α* levels in aortic samples of ApoE^−/−^ mice [114]. Another selective eIF2*α* dephosphorylation inhibitor, salubrinal, protected cells from ER stress by blocking eIF2*α* dephosphorylation [115]. These results suggest a therapeutic strategy for the prevention or treatment of atherosclerosis by means of eIF2*α* phosphorylation inhibitors. Another example is the development of PERK inhibitors. The compound GSK2606414 is an orally available, powerful, and selective first-in-class PERK inhibitor [116]. As a high-affinity ligand of the PERK domain, GSK2606414 inhibits PERK activity by competing with physiological levels of ATP. Furthermore, it was reported that GSK2606414 effectively inhibits PERK-mediated eIF2*α* phosphorylation and protein synthesis regulation in vivo [117]. However, to date, the application of specific small-molecule inhibitors targeting this pathway in the treatment of atherosclerosis still needs further clinical investigation.

There have also been some reports regarding the inhibition of this signaling pathway upregulation in ER stress. Sirtuin 1, an NAD(+)-dependent deacetylase, protected cardiomyocytes against ER stress-induced apoptosis by alleviating activation of the PERK/eIF2*α* branch of the UPR [118]. In addition, a newly discovered myokine that protects against metabolic disorders and atherosclerosis, irisin, was shown to inhibit the PERK/eIF2*α*/CHOP and ATF6/CHOP ER stress signaling pathways, thereby alleviating the apoptosis of cultured RAW264.7 macrophages induced by ox-LDL [119]. Estrogen has a strong antioxidant activity, and its effect on ER stress has been reported. Estrogen significantly inhibited the increase in p-PERK/PERK, p-IRE1/IRE1, and ATF6. In other words, estrogen suppressed ER stress-related apoptosis that was triggered by the PERK pathway by activating the PI3K-Akt pathway to protect HUVECs [120]. In addition, dextrose-induced ER stress and superoxide generation were inhibited in HUVECs by estradiol and interrelated sex steroids [121].

IRE1 (and its downstream effector XBP1) is another important branch of UPR signaling. Targeted regulation of IRE1 is a promising approach for mitigating ER stress and subsequently reversing the progression of atherosclerosis. Treatment of macrophages with IRE1 inhibitors, such as the small molecules STF-083010 and 4*μ*8C, significantly inhibited lipid-induced mitochondrial ROS production, NLRP3 inflammasome activation, and consequent secretion of IL-1 and IL-18, and it reduced T helper type 1 immune responses in ApoE^−/−^ mice [122]. These results indicate that reduced atherosclerotic plaque size caused by IRE1 inhibitors might be mediated by their anti-inflammatory effects, rather than altering plasma lipid profiles [122]. A recent study showed that IRE1*α* plays a crucial protective role in senescent-related ER stress-induced apoptosis, and suppression of IRE1*α* and its downstream effector XBP1 alleviated tunicamycin-induced macrophage apoptosis in older but not younger mice [64]. These results suggest that small molecule IRE1 inhibitors can improve the clinical course of atherosclerosis, independent of the involvement of the CHOP- and JNK-mediated apoptotic pathways, which were the focus of other previous studies [123, 124].

CHOP is a major UPR target of atherosclerosis with very notable potential, and it mediates the major proapoptotic pathways induced by ER stress. However, no selective CHOP inhibitors have been designed to date.

### 4.3. Physiological Inhibitors of ER Stress Targeting AMPK

The AMPK signaling pathway is also implicated in regulating ER stress. Studies have shown that AMPK functions as a physiological inhibitor of ER stress, and this inhibitory effect is achieved by maintaining SERCA activity and intracellular Ca^2+^ homeostasis [113]. By enhancing SERCA oxidation, oxidized and glycated LDL subsequently induces abnormal ER stress, endothelial dysfunction, and atherosclerosis in HFD-fed mice in vivo; all of these conditions were shown to be suppressed by AMPK activation [125]. AMPK is activated by pharmacological drugs such as metformin and statins. Atorvastatin, a widely studied pharmacological compound, was reported to inhibit ER stress through AMPK activation in both atherosclerotic mice and cultured HUVECs [126].

Vascular calcification is an important characteristic of atherosclerosis. The silencing of DAPK3, which is involved in vascular remodeling, alleviated calcification of VSMCs via AMPK-mediated inhibition of ER stress signaling [79]. The peroxisome proliferator-activated receptors (PPARs) were reported to regulate systemic lipid homeostasis and inflammation. Wy-14643, a PPAR-*α* agonist, was found to attenuate the increase in the majority of lipid-induced ER stress markers in human cardiac myocytes by enhancing AMPK activity, which might be beneficial in preventing the harmful influence of ER stress in associated CVDs [127]. Moreover, other AMPK activators, such as PT1 and A-769662, could exhibit protective effects on cardiac myocytes by inhibiting ER stress.

### 4.4. Regulation of ER Calcium Homeostasis

The maintenance of ER calcium homeostasis is another crucial target. Imbalanced calcium homeostasis is an important mechanism for activation of the apoptotic pathway mediated by CHOP in macrophages and the apoptotic pathway mediated by the ER stress-mitochondrial cascade in ECs (see above). Regulation of ER calcium homeostasis, on the one hand, can occur by reducing the efflux of Ca^2+^ from the ER lumen. One example is the use of antihypertensive calcium channel blockers, such as verapamil, to block the Ca^2+^ channel. In diabetic mice, oral verapamil suppressed TXNIP expression and *β*-cell apoptosis and improved glucose homeostasis [128]. However, its role in atherosclerotic cells needs to be studied further. Under ER stress, Ca^2+^ efflux from the ER lumen causes cytosolic Ca^2+^ overload, which in turn activates mitochondrial-mediated apoptosis. Cyclophilin D is necessary for Ca^2+^ influx in the mitochondrial inner membrane, and its inhibitor cyclosporin A can protect cells from ER stress by inhibiting mitochondrial Ca^2+^ influx [129].

Regulation of ER calcium homeostasis, on the other hand, can also occur by increasing SERCA expression and further increasing Ca^2+^ influx [130]. Obesity and insulin resistance have been shown to be activators of ER stress-induced apoptosis [6]. In hyperinsulinemia, the signaling of functional insulin receptors in macrophages was downregulated, which involved the elevation of cytosolic calcium by SERCA inhibition, thus promoting ER stress and apoptosis [131]. An effective target for enhancing SERCA activity and upregulating Ca^2+^ influx is AMPK. As mentioned earlier, AMPK suppresses ER stress by maintaining SERCA activity and intracellular Ca^2+^ homeostasis, and AMPK activation inhibits the reduction in ox-LDL-induced SERCA activity and oxidative enhancement, which lead to ER stress.

### 4.5. Remover of Atherorelevant Inducers of ER Stress

The atherosclerotic inducers of pathological ER stress we described earlier mainly include Hcy and modified LDL. In addition to the Hcy-induced ER stress in several types of atherosclerotic lesional cells mentioned above, Hcy was also found to enhance ER stress in T cells and promote T cell activation and cytokine secretion by increasing ER-mitochondria coupling, thereby accelerating atherosclerosis [132]. As cholesterol-lowering drugs, statins actually have pleiotropic effects. It was found that Hcy-induced ER stress and vascular damage in ApoE^−/−^ mice were inhibited by atorvastatin, and this protective effect was mediated by AMPK activation [107]. Moreover, atorvastatin inhibited Hcy-induced ER stress and downregulated the expression of TNF-*α* and matrix metalloproteinase- (MMP-) 9 mRNA in macrophages, thus improving the stability of atherosclerotic plaques in HHcy mice [133]. ox-LDL-induced endothelial apoptosis is essential for atherosclerosis. Simvastatin can inhibit ER stress and apoptosis induced by ox-LDL in VECs. Exposure of HUVECs to ox-LDL significantly increased apoptosis, accompanied by elevated PERK expression, CHOP mRNA levels, and caspase-3 activity; these effects were all suppressed after simvastatin treatment [41].

### 4.6. Targeting ER Stress by MicroRNAs

MicroRNAs (miRs) have been shown to protect from atherosclerosis by preventing endothelial inflammation and formation of atherosclerotic lesions [134]. The association between ER stress and miRs indicates that the latter may be a new target for atherosclerosis treatment. Inhibition of miR-103 alleviated inflammation and ER stress in atherosclerotic mice by blocking phosphatase and tensin homolog- (PTEN-) mediated mitogen-activated protein kinase (MAPK) signaling [135]. MAPK signaling has been confirmed to participate in atherosclerosis by regulating the proliferation and migration of VECs, and miR-29b downregulation attenuated atherosclerosis by suppressing the MAPK signaling pathway and inflammation in the aortas of ApoE^−/−^ mice [136]. Another example is that miR-107 activated the Notch pathway by targeting keratin 1 (KRT1) gene inhibition, consequently protecting VECs from inflammation and ER stress in a mouse model of coronary atherosclerosis [137].

Previous studies have shown that ER stress regulates cholesterol metabolism through multiple pathways in atherosclerosis. Among them, the upregulation of miR-33 and CHOP activation were confirmed to be involved in the lipid metabolism disorder induced by ER stress in atherosclerotic macrophages [138]. In addition, overexpression of miR-384 inhibited angiotensin II-induced apoptosis and ER stress in HUVECs, which was caused, at least in part, by downregulation of HERP expression [139].

### 4.7. Targeting ER Stress by Natural Compounds

Targeting ER stress by natural products opens an exciting therapeutic window for the treatment of atherosclerosis (Table 1). Some natural ingredients can inhibit the upregulated signaling pathways in ER stress and thus play a protective role in atherosclerosis. Kaempferol, a phytoestrogen, significantly suppressed the expression of GRP78 and CHOP under stress conditions and alleviated ER stress-induced cell death by targeting caspase-3 and caspase-7 [140]. Quercetin, a flavonoid, inhibited the increased expression of CHOP, GRP78, and ATF6, as well as the activation of JNK and caspase-12 in RAW264.7 macrophages, thus preventing glucosamine-induced apoptosis and lipid accumulation through the ER stress pathway [141]. A previous study found that resveratrol, a polyphenol antioxidant found in red wine, effectively inhibited isoproterenol-induced cardiomyocyte hypertrophy and apoptosis partially by suppressing ER stress; this included reducing the expression of GRP78, GRP94, and CHOP proteins and reversing the expression of Bcl-2 and Bax [142]. Another active ingredient, baicalin, from the root of *Scutellaria*, was found to protect cardiac myocytes from ER stress-induced apoptosis by the CHOP/eNOS/NO pathway [143].

As an independent risk factor for atherosclerosis, Hcy can damage VECs through various mechanisms including promoting the oxidative stress and ER stress pathways. It has been reported that salidroside inhibits the activation of BiP/GRP78 and CHOP induced by Hcy, suppresses the phosphorylation of PERK or IRE1*α*, and protects HUVECs from Hcy-induced injury by regulating ER stress [144]. Catalpol, which was extracted from the root of *Rehmannia glutinosa*, was found to inhibit Hcy-induced ROS overgeneration and inflammation by suppressing the GRP78/PERK and NADPH oxidase 4 (Nox4)/NF-*κ*B pathways in human aortic endothelial cells (HAECs) [145].

Ischemia and hypoxia are two other important factors in ER stress induction. Berberine, an isoquinoline-derived alkaloid isolated from Rhizoma coptidis, was shown to ameliorate myocardial ischemia/reperfusion injury and alleviate ER stress-induced apoptosis, which was evidenced by suppression of PERK and eIF2*α* phosphorylation, as well as the expression of ATF4 and CHOP in the rat myocardium. Furthermore, sulforaphane from cruciferous vegetables was shown to effectively downregulate ischemia-enhanced ER stress, autophagy, and apoptosis and subsequently to attenuate ischemia-induced dysfunction in rat bladders [146].

Some natural ingredients have antioxidant, anti-inflammatory, and other beneficial effects that can attenuate ER stress. Curcumin, a natural polyphenolic antioxidant compound, can inhibit the NF-*κ*B signaling pathway and is known for its anti-inflammatory and immunomodulatory effects. Interestingly, however, curcumin has been confirmed to induce apoptosis of activated human CD4^+^ T cells by enhancing ER stress and mitochondrial dysfunction, as evidenced by increased PERK and IRE1 phosphorylation, increased XBP1 and CHOP expression, and decreased expression of the antiapoptotic protein Bcl-2 [147]. In addition, in a HUVEC injury model induced by hyperglycemia, which is a stimulator of atherosclerosis development in diabetes, crocin played an antioxidant, antiapoptotic, and anti-inflammatory role, which might be mediated by modification of ER stress [148].

## 5. Conclusion

ER stress acts as an adaptive and defensive response of the body to harmful stimuli and induces a compensatory protective mechanism by activating the UPR. However, if the ER stress is prolonged or too strong, the UPR can no longer normalize ER function, which leads to the activation of inflammation and proapoptotic signaling pathways in different types of cells in the arterial wall, affecting the formation and vulnerability of atherosclerotic plaques. These factors play a key role in the pathogenesis of many diseases including atherosclerosis and CVDs. A growing number of studies have also confirmed that targeting ER stress and the UPR signaling pathways may be novel strategies for the treatment of atherosclerosis. Herein, we also reviewed the application of chemical chaperones, inhibitors of upregulated UPR signaling pathways in ER stress, regulation of AMPK, some microRNAs with antiatherogenic protective effects, and some natural compounds that target the ER stress pathways. In conclusion, these studies on the role of ER stress in atherosclerosis may lead to the development of novel strategies and directions for the prevention and treatment of atherosclerosis and associated CVDs.

## Figures and Tables

**Table 1:** Natural compounds target endoplasmic reticulum (ER) stress to ameliorate atherosclerosis.

Natural compound	Source and/or chemical class	Effect target or biological function	Effect on ER stress and atherosclerosis	Reference
Kaempferol	Phytoestrogen	↓GRP78 and CHOP expression; targeting caspase-3/7	Alleviates ER stress-induced cell death	[140]
Quercetin	Flavonoid	↓CHOP and GRP78 expression; activated JNK and caspase-12; ↑ATF6 expression	Prevents glucosamine-induced apoptosis and lipid accumulation by inhibiting ER stress in RAW264.7 macrophages	[141]
Resveratrol	Polyphenol antioxidant found in red wine	↓GRP78, GRP94, and CHOP expression; reversing the expression of Bcl-2 and Bax	Effectively inhibits isoproterenol-induced cardiomyocyte hypertrophy and apoptosis partially by suppressing ER stress	[142]
Baicalin	From the roots of *Scutellaria*	Targeting the CHOP/eNOS/NO pathway	Protects cardiac myocytes from ER stress-induced apoptosis	[143]
Salidroside	Active component of *Rhodiola rosea*	↓BiP and CHOP activation; ↓PERK or IRE1*α* phosphorylation	Protects HUVECs from Hcy-induced injury by regulating ER stress	[144]
Catalpol	Extracted from *Rehmannia glutinosa* root	↓GRP78/PERK and Nox4/NF-*κ*B pathways	Attenuates Hcy-induced ROS overgeneration, inflammation, and cell apoptosis in HAECs	[145]
Sulforaphane	From cruciferous vegetables	Regulating expression of GRP78 and CHOP, autophagy-related Beclin-1, p62, and LC3-II, and apoptosis caspase-3 pathway	Effectively reduces ischemia-enhanced ER stress, autophagy, and apoptosis	[146]
Curcumin	Natural polyphenolic antioxidant compound	↓NF-*κ*B signaling pathway; ↑PERK and IRE1 phosphorylation; ↑XBP1 and CHOP expression; ↓anti-apoptotic protein Bcl-2	Enhances ER stress and mitochondrial dysfunction, thus inducing apoptosis of activated human CD4^+^ T cells	[147]
Crocin	Main ingredient of saffron	Plays antioxidant, antiapoptotic, and anti-inflammatory roles	Protect HUVECs from high glucose-induced injury by suppressing ER stress response	[148]

HAECs: human aortic endothelial cells; Hcy: homocysteine; eNOS: endothelial nitric oxide synthase; ROS: reactive oxygen species.
